# The role of neo-adjuvant therapy in cholangiocarcinoma: A systematic review

**DOI:** 10.3389/fonc.2022.975136

**Published:** 2022-12-08

**Authors:** Sinead Cremen, Michael E. Kelly, Tom K. Gallagher

**Affiliations:** ^1^ Department of Hepatobiliary Surgery, St. Vincent’s University Hospital, Dublin, Ireland; ^2^ Department of Surgery, Tallaght University Hospital, Tallaght, Dublin, Ireland

**Keywords:** cholangiocarcinoma (CC), neoadjuant chemotherapy, outcomes, review – systematic, surgery, resection

## Abstract

**Introduction:**

Cholangiocarcinoma (CCA) is the most common malignancy affecting the biliary tree. The only curative treatment is surgical resection, aiming for negative margins (R0). For those who have locally advanced disease, which is borderline resectable, neoadjuvant chemoradiation presents an opportunity to reduce tumour size and allow for surgical resection. The aim of this review is to establish the role of neoadjuvant therapy in each subtype of CCA and establish its impact on survival.

**Methods:**

Search terms such as ‘neoadjuvant therapy’ and ‘cholangiocarcinoma’ were searched on multiple databases, including Pubmed, Ovid and Embase. They were then reviewed separately by two reviewers for inclusion criteria. 978 studies were initially identified from the search strategy, with 21 being included in this review.

**Results:**

5,009 patients were included across 21 studies. 1,173 underwent neoadjuvant therapy, 3,818 had surgical resection alone. 359 patients received Gemcitabine based regimes, making it the most commonly utilised regimen for patients CCA and Biliary Tract Cancer (BTC). Data on tolerability of regimes was limited. All included papers were found to have low risk of bias when assessed using The Newcastle Ottawa Scale. Patients who underwent neoadjuvant therapy had a similar median overall survival compared to those who underwent upfront surgery (38.4 versus 35.1 months respectively). Pre-operative CA19-9, microvascular invasion, perineurial invasion and positive lymph nodes were of prognostic significance across BTC and CCA subtypes.

**Conclusion:**

Neoadjuvant therapy and surgical resection is associated with improved patient outcomes and longer median overall survival compared to therapy and upfront surgery, however heterogeneity between research papers limited the ability to further analyse the significance of these results. Although initial studies are promising, further research is required in order to define suitable treatment protocols and tolerability of neoadjuvant regimes.

**Systematic review registration:**

https://www.crd.york.ac.uk/prospero/, identifier CRD42020164781.

## 1 Introduction

Cholangiocarcinoma (CCA) is the most common malignancy affecting the epithelium of the biliary tree and the second most common hepatic malignancy (1, 2). Incidence varies between regions, with rates of 0.5-2.0 per 100,000 in western countries up to 100 per 100,000 in Thailand (3). It can be classified anatomically as intrahepatic CCA (iCCA), hilar CCA (hCCA) and distal CCA (dCCA) (4). hCCA accounts for 50% of cases, dCCA accounts for up to 40%. iCCA is the rarest subtype of cholangiocarcinoma, making up the remaining 10% (5, 6).

Patients with cholangiocarcinoma can present with variable symptoms depending on the location. dCCA and hCCA most commonly present with jaundice (7). Prior to the onset of jaundice, symptoms are non-specific, including fatigue, weight loss, abdominal pain and night sweats (7, 8). Although a number of risk factors for CCA are known, including primary sclerosing cholangitis (9, 10), many patients have no apparent risk factors at diagnosis (10). Owing to vague early symptoms and no identifiable risk factors, patients often present with advanced disease, with up to 25% of iCCA diagnosed incidentally (7, 11).

As the only curative management to date is surgical resection with clear margins (R0) (2, 12), the extent of tumour spread is important with regard to potential surgical management. As few as 30% of patients are deemed to have resectable disease at diagnosis (3, 5). A further 10-45% of patients who are initially deemed to have resectable disease are found to be unresectable at exploration (1). Even with surgical resection 5 year survival is limited, ranging from 23-44% (2, 13, 14).

Neoadjuvant chemoradiation therapy has become a mainstay of management for patients with locally advanced rectal and breast cancer (15, 16)., and there has been promising results in pancreatic cancer (17). It has been hypothesized that it may be beneficial in CCA, particularly for patients who present with borderline resectable or locally advanced disease. Initial success was seen in this with the development of the Mayo Protocol, consisting of neoadjuvant chemo-radiation therapy followed by Liver Transplant for management of hCCA. They demonstrated favourable long term survival (18, 19) which has been replicated in other centres (20).

This review aims to systematically evaluate the existing literature regarding the role of neoadjuvant therapy in the management of cholangiocarcinoma in order to assess the survival outcomes conferred by neoadjuvant therapy for each cholangiocarcinoma subtype.

## 2 Methods

### 2.1 Search strategy

This study was registered with PROSPERO (registration number CRD42020164781) prior to starting and was carried out using PRISMA guidelines. A search for relevant articles was carried out using the PubMed, Embase, Cochrane, Web of Science and TRIP databases up until February 2021. The search was repeated in June 2022. Key words and subject heading (MeSH) were used, including ‘cholangiocarcinoma’, ‘neoadjuvant therapy’, ‘malignancy’ and ‘neoplasm’.

### 2.2 Inclusion and exclusion criteria

Studies deemed suitable for inclusion were those investigating neoadjuvant therapy in cholangiocarcinoma. Studies were excluded if not published in English, were reviews, editorials, case studies or opinion pieces, if patients only received therapy after surgery or did not undergo surgical resection.

### 2.3 Screening

Title and abstract were reviewed independently for inclusion criteria by two reviewers. Any disagreements were resolved with discussion. Papers identified from title and abstract were then reviewed in full, and 21 papers were included for the final systematic review.

### 2.4 Data extraction

Data extracted included title, journal, year of publication, author, CCA type, number of participants, neoadjuvant regime, surgical procedure, overall survival (OS), disease free survival (DFS), and other clinical outcomes investigated. OS and DFS were extracted from the studies included and were from time of diagnosis. A standardised form was developed so that data extraction was standardized between papers and reviewers. Data was reviewed and extracted by two independent reviewers. Resectable, locally advanced, borderline resectable and non- resectable were identified in each paper. Thus, definitions of each were somewhat variable, but resectable cancer was mostly defined as those curable with surgical resection. Locally advanced were cancers with local invasion, such as localized liver metastasis or lymph node disease. Borderine resectable disease were cases with locally advanced disease that would be resectable with extensive surgical disease. Non-resectable were defined as those that would not be curable with surgical resection.

### 2.5 Bias

Both reviewers independently assessed for bias using The Newcastle Ottawa Scale (NOS). As per the NOS, if a study reaches 7 points, there is a low risk of bias. When comparing extracted data for papers, NOS scores were also reviewed to ensure similar NOS score was awarded to each study.

### 2.6 Statistical analysis

Due to the limited number of papers identified and heterogeneity of data, it was deemed inappropriate to carry out a meta-analysis. Median OS and DFS were calculated by identifying documented OS and DFS in each study and identifying the median value.

## 3 Results

### 3.1 Search results

**Figure 1:**
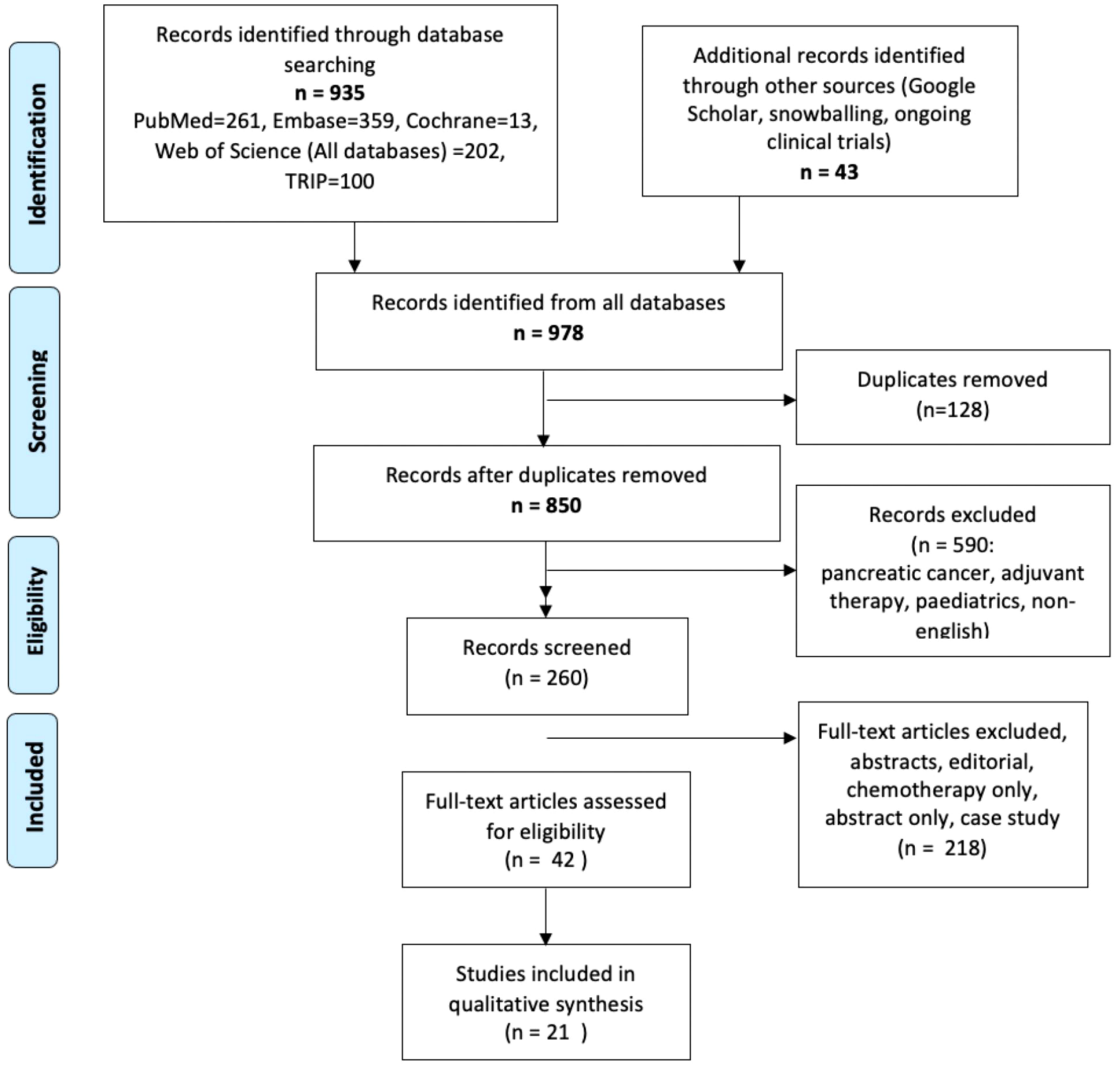
PRISMA Flow Chart demonstrating papers identified in the search and those included in the final review.

978 papers were initially identified from the search (**Figure 1**). 128 duplicates were excluded and 590 papers were excluded as they did not reach the inclusion criteria. 21 studies were eligible for inclusion. 15 studies were retrospective cohort studies and the remainder were single centre prospective studies. One paper reached inclusion criteria, but was published in 1997, included a small number of patients and did not clearly define indications for neoadjuvant therapy so it was excluded (21). Seven papers investigated the role of neoadjuvant therapy in the management of biliary tract cancers, which include CCA, gallbladder cancer and ampullary cancer. Six assessed iCCA alone, six assessed hCCA and one assessed dCCA. One paper did not identify any CCA subtype (22). (**Table 1**) All studies received an NOS score of greater then 7.

**Table 1:** Study Characteristics.

	Year	Journal	CCA	Study type	Participants	Number receiving NT	Number proceeding to surgery	Neoadjuvant regime	Surgical Procedure	R0 resection	Median OS (months)	Median DFS (months)
(23)	2014	HPB	hCCA (Mayo protocol)	Retrospective	20	20	20	brachytherapy (7.5Gy), external beam radiation (45-55Gy), 5-FU, maintenance- capecitabine	Liver Transplant			37
(24)	2016	Am. J. Surg. Path.	hCCA (Mayo protocol)	Retrospective	152	152	152	EBRT (4000-6000Gy) & 5FU followed by brachytherapy (1000-1500Gy). Maintenance cepecitabine	Liver Transplant			
(25)	2015	Int. J. Mol. Sci.	hCCA (Mayo protocol)	Phase II pilot study	7	7	7	Photodynmaic therapy (haematoporphyrin and laser light irradiation)	Major resection (6) Liver Transplant (1)		38.4	37.2
(26)	2014	Liver Transplantation	hCCA (Mayo protocol)	retrospective	31	23	5	stereotactic body radiation therapy- 50-60Gy, capecitabine while awaiting LT	Liver Transplant			
(27)	2017	World J. Gastrointest. Surg	hCCA	retrospective	57	12	12	5FU, leucovorin (5), gemcitabine (5), gemcitabine and cysplatin (1), Tegfur/Uracil (1). All had EBR (40-50Gy)		10	32.9	26
(28)	2020	BMC Cancer	hCCA	Single centre prospective cohort study	72	47	31	No data				
(29)	2017	BJS	iCCA	Single centre prospective cohort study	186	74	33	gemcitabine plus oxaliplatin, ± transarterial chemotherapy/radioembolisation, 19 received other regimes (fluorouracil, oxaliplatin and irinotecan)	liver resection	12	24.1	14·4
(30)	2017	J. Surg. Oncol.	iCCA	Prospective multi-centre cohort	1057	62	55	intra-arterial chemotherapy (18), systemic (44)	Resection	42	46.9	34.1
(31)	2019	JAMA Surgery	iCCA	Retrospective, multi-institutional database study	687	66		No data	resection			
(32)	2017	Surgery	iCCA	Retrospective study from prospectively maintaiend database	119	43		gemcitabine and cisplatin (n = 34), gemcitabine (n = 1), gemcitabine and other chemotherapeutic agent (n = 4), fluorouracil-based chemotherapy (n = 2), taxotere-based chemotherapy including bevacizumab (n = 2)	resection			
(33)	2\020	Ann. Surg. Oncol.	iCCA	Retrospective cohort study of proscpective database	169	32	32	Cisplatin based (17), Gemcitabine and oxaliplatin (10), folfirinox (1), SIRT (19)	Major hepatectomy (31)		45.9	18.5
(34)	2021	Ann. Surg. Oncol.	iCCA	Propensity score matched analysis from public database	881	74	74	Multi-agent regimes		48	51.8	
(35)	2019	Am. J. Surg.	dCCA	Retrospective cohort study	45	21	21	5-FU or gemcitabine based, with radiation (hyperfractionated 30Gy or normal 43-60Gy)	Whipples	19	40.3	
(22)	2019	Eur. J. Surg. Oncol.	CCA	Propensity score matched analysis from public database	978	278	700	multi-drug neoadjuvant chemotherapy (158)		198	40.3	40.3
(36)	2018	J. Gastrointest. Oncol.	GBC, CCA	Retrospective cohort study	128	77	77	chemotherapy (single or multiple agent) ± radiation thereapy	resection		31.2	
(37)	2016	Anticancer Res.	BTC	Single centre retrospective study	339	44	44	5-FU or tegafur and uracil or gemcitabine in combination with 50-60Gy of radiation	Resection or whipples			
(38)	2017	Eur. J. Surg. Oncol.	BTC	Single centre retrospective study	194	27	27	gemcitabine and 50-60Gy radiation	Resection or whipples			
(39).	2012	Ann. Surg. Oncol.	BTC	Single centre retrospective study	22	22	22	gemcitabine	resection	4		
(40)	2015	Ann. Surg. Oncol.	BTC	Prospective case control with hidtoruc group	39	39	39	gemcitabine ± cisplatin	resection	7	17.8	
(41)	2015	Cancer Chemother. Pharmacol.	BTC	Phase I study	25	25	25	gemcitabine combination radiation therapy	resection	24		
(42)	2013	J. Gastrointest. Surg.	BTC	Retrospective study from prospective cohort	157	28	28	gemcitabine/platinum-based chemotherapeutics or 5-fluorouracil	resection		42.3	

iCCA, intrahepatic cholangiocarcinoma; hCCA, hilar cholangiocarcinoma; dCCA, distal cholangiocarcinoma; CCA, cholangiocarcinoma; BTC, biliary tract cancer; OS, overall survival; DFS, disease free survival; NT-S, neoadjuvant therapy and surgery; S, surgery alone; 5-FU, 5 flurouracil; LT, liver transplant; SIRT, selective internal radiation therapy; Gy, grey.

### 3.2 Type of cholangiocarcinoma

1,173 (23%) of 5,009 patients identified underwent neoadjuvant therapy, while 3,818 (76%) had surgical resection alone and 18 patients were excluded due to metastatic disease. Following neoadjuvant therapy, 1,142 (85%) underwent surgical resection. 351 (30%) of those who underwent neoadjuvant therapy had a diagnosis of iCCA, 261 (20%) had hCCA, 21 (19%) had dCCA, 383 (33%) had CCA and 157 (13%) had BTC (CCA and gallbladder cancer) (**Table 2**). In comparison, 2732 of those who immediately underwent surgical resection had iCCA (72%), 103 had hCCA (3%), 24 had dCCA (0.6%), 700 had CCA (18%) and 259 had BTC (7%).

**Table 2:** Demographic Information.

	Neoadjuvant Therapy and Surgery	Surgery
**Age (median)**	62	64.5
**iCCA**	351	2732
**hCCA**	261	103
**dCCA**	21	24
**CCA**	383	700
**BTC**	157	259
**Adjuvant (n=)**	128	1119
**OS (months)**	38.4	32.8
**DFS (months)**	26	15.1

iCCA, intrahepatic cholangiocarcinoma; hCCA, hilar cholangiocarcinoma; dCCA, distal cholangiocarcinoma; CCA, cholangiocarcinoma; BTC, biliary tract cancer; OS, overall survival; DFS, disease free survival.

### 3.3 Neoadjuvant treatment

Most patients were referred to neoadjuvant therapy due to locally advanced disease (29, 33), Welling et al. also referred patients to neoadjuvant therapy if there was an underlying diagnosis of PSC, regardless of stage (43). One study of 278 did not identify the neoadjuvant therapy received, only stating it was multi-agent therapy regimes (22). Two studies referred all patients to neoadjuvant therapy (30, 41). 3 studies determined if patients received neoadjuvant therapy based on tumour resectability on radiological imaging (27, 28, 32). Two groups used a combination of CT imaging, clinical exam, CA19-9 and biopsy results to determine if neoadjuvant chemotheraoy was required (29, 43). One study determined treatement depending on projected remaining liver volume (38). One group referred patients to neoadjuvant therapy due to unresectable disease (33). 8 studies did not identify a reason for referring patients onto neoadjuvant therapy (22–25, 31, 34–36).

#### 3.3.1 iCCA

351 iCCA patients were referred to neoadjuvant therapy, with 133 (38%) receiving unspecified regimes, Gemcitabine based regimes in 100 (29%), transarterial therapy or radioembolization in 4 (1%), Selective Internal Radiation Therapy (SIRT) in 19 (5), 5-FU in 2 (0.5%) and other regimes (including oxaliplatin, cisplatin, irinotecan, tegafur, uracil and Taxotere) in 39 (11%) (29–34, 42).

#### 3.3.2 hCCA mayo protocol

Of the 261 patients included with hCCA, 202 (77%) were managed with the mayo protocol or a similar variant. 179 (88%) were started on 5-FU as part of the Mayo protocol. 12 (6%) underwent stereotactic body radiation therapy (SBRT) without therapy. 35 adverse effects were recorded, 14 of which were classified as significant adverse effects (43). Cholangitis was the most common significant adverse effect. 191 (95%) underwent external beam radiation therapy (EBRT) in addition to therapy. All patients undergoing the Mayo Protocol were prescribed maintenance capecitabine. A total of 4 patients required a dose reduction of capecitabine due to adverse effects (43).

#### 3.3.3 hCCA

59 patients with hCCA did not undergo the Mayo protocol. They received gemcitabine (33), 5FU and leucovorin (5), gemcitabine and cysplatin (1) or tegfur and uracil (1) (27, 28). 7 patients underwent photodynamic therapy, which consisted of haemoporphyrin and laser light therapy (25). One study reported 49% of patients undergoing neoadjuvant therapy experienced grade 3-4 toxicity (28).

#### 3.3.4 dCCA

21 dCCA patients underwent preoperative therapy, with 9 (42%) receiving gemcitabine based regimes, 2 (9.5%) receiving 5-FU based regimes (35). 16 (76%) patients underwent EBRT. No information was identified regarding tolerability of the different regimes (35). Neoadjuvant therapy was carried out due to concerns regarding advanced disease (9), performance status (7) or a possible diagnosis of pancreatic ductal adenocarcinoma (3) (35).

#### 3.3.5 BTC

Of the 185 patients who were referred on to neoadjuvant therapy, 72 (39%) received 5-flurouracil, tegafu, uracil or gemcitabine base regimes. 74 (40%) received gemcitabine alone. While 39 (21%) received gemcitabine with cisplatin. Chemotherapy regime was unspecified in 69 cases (37%). 14 (7.5%) underwent radiation therapy without chemotherapy while 91 (49%) had radiation in addition to chemotherapy. 21 of 25 patients who received gemcitabine and EBRT experienced adverse effects, which included deranged Liver Function Tests (22), leukopenia (11), biliary stent changes (11), thrombocytopenia (8), and constipation (6) (41).

### 3.4 Resection margins

Resection margins were examined in 12 papers (22, 23, 25– 27, 29, 30, 32, 34– 36, 42). However, for hCCA (25), dCCA (30) and CCA (22), only one study was examined respectively. In those who underwent neoadjuvant therapy, the R0 resection rate ranged from 31% to 90%, compared to 29% to 83%

#### 3.4.1 iCCA

R0 resection varied from 31% to 73% in the neoadjuvant group and 49.6% to 87% in surgery alone. Those who underwent neoadjuvant therapy had increased rates of R1 (neoadjuvant therapy- 24%-67% vs. surgery alone 5%-48.8%) and R2 resection (1.75%-3% neoadjuvant vs. 0.41%-1.7% surgery alone) (29, 30, 32–34).

#### 3.4.2 hCCA

R0 resection was achieved in 17 (37%) patients who underwent neoadjuvant therapy, compared to 30 (29%) of those who went straight to surgery (25, 27). On histology, perineural invasion was identified in 12 (26%) of neoadjuvant samples and 44 (43%) of surgery alone samples (27).

#### 3.4.3 dCCA

19 (90%) of patients who received neoadjuvant therapy prior to surgery had an R0 resection. In comparison, 20 patients (83%) of those who had surgery alone had an R0 resection. Lymphovascular invasion was found on histology on 14 (58%) and 7 (33%) of those who had surgery alone and neoadjuvant therapy respectively. Perineural invasion was found on 16 (66%) who had surgery and 11 (52%) who received neoadjuvant therapy (35).

#### 3.4.4 CCA

R0 resection was increased in those who underwent neoadjuvant therapy when compared to adjuvant. 198 (71%) of those who received neoadjuvant had R0 resection, compared to 428 (61.1%) (22).

#### 3.4.5 BTC

Resection margins were reported in 87 cases, with 70 (80%) achieving R0 resection (37, 39–41). However 28 were initially resectable BTC who underwent neoadjuvant herapy (37).

### 3.5 Post-operative complications

The rate of post-operative morbidity ranged from 12% to 75% in patients undergoing neoadjuvant therapy (27, 30, 33, 37, 39, 41, 42), compared to 23-39% in those who had surgery alone (27, 30, 33). There was no significant difference between grade III-IV Clavien-Dindo complications between the two groups (29, 30). However, several studies did not compare Clavien-Dindo graded complications, but rather the type of complication (27, 39, 40, 42). No data was included on post-operative course for patients with a diagnosis of dCCA or CCA alone (22, 35).

#### 3.5.1 iCCA

71 (20%) patients experienced post-operative complications. The neoadjuvant therapy group had significantly increased incidence of post-operative complications (Neoadjuvant therapy 59% vs surgery alone 39%, p=0.002) and increased risk of readmission (neoadjuvant therapy 15.7% vs surgery alone 4.8%, p=0.001) (17). There was no difference in Clavien-Dindo grade 3-4 complications (29, 30), hospital length of stay (LOS) (29) or post-operative mortality (29) between the two groups. Merath et al. investigated factors affecting ‘textbook outcomes’ in surgical resection of iCCA, defined as a composite measure of postoperative outcomes including margin status, perioperative infusion, postoperative infection, LOS and readmission or mortality within 30 days post-operatively. 66 (13%) of patients in this study received neoadjuvant therapy and they were more likely not to have a favourable textbook outcome (p=0.001) (18).

#### 3.5.2 hCCA mayo protocol

11 transplant related complications and four post-operative death occurred post OLT. Five patients died between 4 to 109 days post-transplant (23, 43). Two of these required re-transplantation due to hepatic artery thrombosis and primary graft non-function. Cause of death included cardiac arrest (2), disseminated intravascular coagulopathy (1), hepatic artery thrombosis (1) and pancreatic leak and haemorrhage post retransplant (1). Two patients required surgery due to bleeding secondary to pancreatic leak (43).

#### 3.5.3 hCCA

Six complications were documented in patients receiving neoadjuvant therapy (27). The most common complication was bile leaks, seen in two patients. Other complications included small bowel obstruction (1), wound seroma (1), subdiaphragmatic haematoma (1) and pancreatic insufficiency (1) (27).

#### 3.5.4 BTC

Three papers reported post-operative complications (n=79). The most common complication was pancreatic fistula, seen in 13 cases (16%), followed by biliary leak in 8 (10%). Kobayashi et al. found that those with initially unresectable CCA had a higher incidence of severe biliary leak (37). Other complications included abdominal abscess (7), biliary fistula (4), hyperbilirubinaemia (3), haemorrhage (2), cholangitis (2), delayed gastric emptying (2), ascites (2), liver abscess (1) and MRSA wound infection (1) (37, 39, 41).

### 3.6 Disease free survival

The median disease free survival (DFS) ranged from 7.2 to 37 months (23, 25, 27, 29, 30, 33). Compared to those who underwent surgery alone, those who received neoadjuvant therapy had a marginally longer DFS (27, 29, 30, 38).

#### 3.6.1 iCCA

Patients who underwent neoadjuvant therapy had a longer DFS compared to those who went straight to surgery (7.2-34.1 months versus 11.8-29.1 months respectively) (29, 30, 33). One group found that there was a similar DFS in neoadjuvant therapy versus surgery alone, but those who received pre-operative SIRT had a significantly longer DFS then those who had surgery alone (18.5 months versus 11.3 months respectively) (33). Omichi et al. found that DFS was independently associated with neoadjuvant therapy, neutrophil to lymphocyte ratio <3 and elevated pre-treatment CEA (32).

#### 3.6.2 hCCA mayo

Lehrke et al. reported a 5 year DFS of 74%, with a total of 35 of 152 patients experiencing disease recurrence (24). In a study of 20 patient who completed the Mayo Protocol, nine were disease free a median of 37 months post OLT, while the remaining patients either died secondary to complications (4) or due to disease recurrence (7) (23).

#### 3.6.3 hCCA

Median DFS was longer in those who underwent neoadjuvant therapy prior to surgery then those who underwent surgery alone, 26-37.1 months and 15.1 months respectively (25, 27).

#### 3.6.4 BTC

No group described median DFS in the BTC group. 3 and 5 year DFS was higher in those receiving neoadjuvant therapy. 3 year DFS was 78% in the neoadjuvant group compared to 57% in the surgery alone group. At 5 years, DFS was 78% and 52% respectively (38).

### 3.7 Overall survival

OS was reported in 19 papers and sincluded all those who underwent neoadjuvant therapy regardless of the extent of disease. There was a median of 38.4 months in those who underwent neoadjuvant therapy and 32.8 months in those who underwent surgical resection alone. Those who underwent chemotherapy alone had a shorter median survival, at 8.5 months, compared to neoadjuvant chemotherapy and surgery (29, 39, 40).

#### 3.7.1 iCCA

Median OS was reported in two studies and ranged from 24.1 to 51.8 months in those receiving neoadjuvant therapy compared to 25.7 to 37.4 in patients who did not have preoperative therapy (29, 30, 33, 34). Subgroup analysis demonstrated neoadjuvant therapy was associated with significantly longer OS in those with stage II or III disease (34). Median OS in those who underwent chemotherapy alone was shorter again, at 7.8 months (29).

#### 3.7.2 hCCA mayo protocol

Median OS could only be established in one study, at 35.5 months (23). One year OS ranged from 75% to 83% (23, 43), while 5 years OS was 69% in a study of 152 patients (24).

#### 3.7.3 hCCA

Median OS was 32.9 months and 27.1 months, for neoadjuvant and surgery alone respectively (25). For those who received photodynamic therapy preoperatively median 1 year OS was 86%, while 5-year OS was 43%.

#### 3.7.4 dCCA

Median OS in those who had neoadjuvant therapy and *de novo* surgery was 40.3 months and 50.3 months respectively. Although those who had surgery alone had a longer OS, the range for both was similar, at 0-115 months and 0-101.8 months for neoadjuvant therapy and surgery respectively (35).

#### 3.7.5 CCA

A significantly longer OS was noted in those who underwent neoadjuvant therapy compared to adjuvant chemotherapy, 40.3 compared to 32.8months in the neoadjuvant group and adjuvant group respectively (22). A number of factors were identified on multivariate analysis which contributed to a longer OS in neoadjuvant therapy, including age group 18-54 years, male, Charlson Comorbidity Index 1-2, intrahepatic tumour and stage one disease.

#### 3.7.6 BTC

Overall survival in patients with BTC varied from 17.8 to 42.3 months in those receiving neoadjuvant therapy to 31.2 to 53.5 in surgery alone (36, 39, 40, 42). Neoadjuvant therapy alone had a lower OS, ranging from 8.5 to 12.4 months (39, 40). Only one study found OS to be higher in those who underwent surgery first, with an OS of 52.5 months compared to 42.3 months in neoadjuvant recipients (42).

### 3.8 Prognostic factors

#### 3.8.1 iCCA

Positive lymph nodes were found to be the only significant prognostic factors for both OS and DFS in patients with iCCA on multivariate analysis (29). However, Riby et al. reported increasing age, extent of hepatectomy and increased size of tumour were also poor prognostic indicators (33).

#### 3.8.2 hCCA mayo protocol

There was an association between risk of recurrence and residual tumour, increased CA19-9 pre neoadjuvant therapy, perineural and lymphovascular invasion (23, 24).Those with both perineural invasion and lymphovascular invasion were found to be 16.3 times more likely to have recurrence (24).

#### 3.8.3 dCCA

Lymph node status was found to be the strongest predictor of survival in both groups. Pre and postoperative therapy were found to be a positive prognostic indicator on multivariate analysis (35).

#### 3.8.4 BTC

Neoadjuvant therapy, arterial invasion and lymph node invasion on CT imaging were each identified to be independently associated with DFS (37). Margin status were found to be a significant indicator for long term survival, with R0 resection conferring a median survival of 5.6 years (42).

## 4 Discussion

Those with unresectable CCA face dismal outcomes with chemotherapy alone. With neoadjuvant therapy, borderline resectable disease can potentially be reduced to a status whereby R0 resection can be achieved. Current guidelines recommend surgical resection for TNM 1 and 2, but no advice is given regarding the role of neoadjuvant therapy except in the case of hCCA which meet the criteria for liver transplantation (44, 45). By pooling results, this review achieved a clear overview of the literature investigating the role of neoadjuvant therapy in CCA, particularly the impact of neoadjuvant therapy on outcomes.

R0 resection was improved by neoadjuvant therapy in dCCA and hCCA, and was similar in iCCA between those who received neo-adjuvant therapy and those who underwent surgery alone. Lymph node status and local lymphovascular and perineural invasion were prognostic factors in all groups. Lymph node status was a significant prognostic indicator in iCCA for both DFS and OS, strengthening the argument for lymph node sampling and consequent improved tumour staging. This is in accordance with a 2015 international expert consensus which also identified lymph node status as an indicator of prognosis and which recommended lymphadenectomy be carried out at resection (14, 46).

Overall survival was increased by neoadjuvant therapy from a median of 8.5 months (chemotherapy alone) and 35.1 months (upfront surgery) to 38.4 months (neo-adjuvant approach), although it is important to note that prognosis of subtypes of CCA and GBC can be variable. It is important to note that patients were referred to neoadjuvant therapy due to locally advanced disease or poor performance status, this implying a real-life benefit from neoadjuvant therapy. Cambridge et al. also demonstrated a favourable 1 and 5 year survival in patients undergoing OLT for hCCA, in a pooled meta-analysis (47). hCCA is unique, as neoadjuvant therapy has been established as part of the Mayo Protocol (45). No other CCA subtype has a defined treatment protocol for neoadjuvant therapy. Indeed, treatment protocols were not described in 46% of cases in this review. Future studies should describe diagnostic algorithms and treatment protocols in detail.

Treatment regimens employed were mostly antimetabolite and alkylating agent based, which were similar to first line therapies for advanced biliary tract cancer (gemcitabine and cisplatin) (48, 49). In recent years, the prospect of targeted therapy for advanced CCA has been explored, with a number of potential mutations identified including FGFR2, IDH1, BRAF, MSI-H and Vascular Epidermal Growth factor A (VEGF), mTOR pathway inhibitors amongst others (8, 50–54). Although current data is limited regarding the impact of targeted therapies in CCA. However, it is an exciting prospect worthy of further research, given the limited survival of both resectable and unresectable CCA. To date, liver transplantation has been established *via* the Mayo Protocol following neoadjuvant therapy in hCCA. The International Liver Transplantation Society (ILTS) Transplant Oncology Consensus Conference Working Group have recognised the potential role for liver transplantation following neoadjuvant therapy in select patients with iCCA (55).

Although data is lacking in regards to complications rates, those who undergo neoadjuvant therapy may be at increased risk of post-operative complications, particularly in regards to biliary and pancreatic leaks. Two studies found Clavien-Dindo grade 3 or higher complications were similar between groups in regards to iCCA (29, 30). A retrospective study of 54 patients with dCCA found 16.67% of patients experienced major complications following pancreaticoduodenectomy (56). Further analysis into the impact of neoadjuvant therapy on post-operative outcomes, particularly in the context of major complications, is required before a conclusion can be drawn.

The limitations of the data identified require acknowledgment. These include the heterogeneity regarding resectability, CCA subtype diagnosis and treatment protocols. Seven papers examined BTC collectively, which included all CCA subtypes and GBC. Given the heterogeneity between papers, particularly regarding treatment strategies, were highly variable and thus direct comparison was difficult. The heterogeneity between studies meant it was not appropriate to carry out a meta-analysis of the data. There was also potential selection bias, as many of the papers were retrospective meaning a higher proportion of patients proceeded from neo-adjuvant therapy to surgery. Prospective studies are warranted in this area which, like in pancreatic adenocarcinoma, will necessitate strict definitions of non-resectability and borderline resectability, protocolised neo-adjuvant regimens, radiologically and surgically consensus as to what defines a response to treatment, and detailed post treatment follow-up.

In conclusions, there are improved outcomes associated with neoadjuvant therapy in the management of CCA, particularly in patients who have initially unresectable disease. Neoadjuvant therapy confers a longer overall and disease-free survival, comparable to that of surgery alone. Although initial studies are promising, further research is required in order to define suitable treatment protocols and tolerability of neoadjuvant regimes.

## Data availability statement

The original contributions presented in the study are included in the article/supplementary material. Further inquiries can be directed to the corresponding author.

## Author contributions

SC, TG contributed to conception and design of the study. SC and MK carried out the review of papers and MK performed the statistical analysis. SC wrote the first draft of the manuscript. MK and TG reviewed the draft and assisted with subsequent drafts wrote sections of the manuscript. All authors contributed to the article and approved the submitted version.
